# Reconstructing normality following the diagnosis of a childhood chronic disease: does “rare” make a difference?

**DOI:** 10.1007/s00431-017-3085-7

**Published:** 2018-01-16

**Authors:** Evi Germeni, Isabella Vallini, Mario G. Bianchetti, Peter J. Schulz

**Affiliations:** 1 Health Economics and Health Technology Assessment (HEHTA), Institute of Health and Wellbeing, University of Glasgow, 1 Lilybank Gardens, Glasgow, G12 8RZ UK; 2 Institute of Communication and Health (ICH), Faculty of Communication Sciences, Università della Svizzera Italiana, 6900 Lugano, Switzerland; 3 Pediatric Department of Southern Switzerland, Ospedale San Giovanni, 6500 Bellinzona, Switzerland; 4 Faculty of Biomedical Sciences, Università della Svizzera Italiana, 6900 Lugano, Switzerland

**Keywords:** Rare diseases, Bartter syndrome, Celiac disease, Illness experience, Family caregivers, Qualitative research

## Abstract

Living with a childhood chronic disease can be challenging, especially if the diagnosis involves a rare condition. This study sought to elucidate how the diagnosis of a rare disease, as compared to a common, chronic condition, may influence maternal experiences of childhood illness. We conducted face-to-face, semi-structured interviews with 26 mothers of children treated in a pediatric hospital in the province of Lecco, Italy. Half of the participants had a child diagnosed with Bartter syndrome (BS), and the rest had a child suffering from celiac disease (CD). Interviews were recorded, transcribed, and analyzed using an inductive thematic approach. We identified three main themes from the analysis of our data: (1) disrupted normality and the need to know, (2) reconstructing normality, and (3) acting “normal.” Although most participants experienced the disclosure of diagnosis as a relief, processes that facilitated normality reconstruction in celiac families, notably access to appropriate information, social support, and personal contact with comparison others, were found to be important stressors for mothers living with BS.

*Conclusion*: This comparative qualitative study provides evidence on how well-known problems associated with the rarity of childhood diseases impact on families’ efforts to cope with the illness and regain a sense of normality.

**Table:** 

**What is Known:** *• Families living with a rare disease have been found to experience a range of common problems, directly linked to the rarity of these pathologies.*
**What is New:** *• Maximization of both emotional and instrumental social support, through provision of appropriate information or establishment of disease-specific support groups, could greatly contribute to rare disease families’ efforts to cope with childhood illness and regain a sense of normality.*

**Electronic supplementary material:**

The online version of this article (10.1007/s00431-017-3085-7) contains supplementary material, which is available to authorized users.

## Introduction

Living with a childhood chronic disease can be challenging, especially if the diagnosis involves a rare condition [11]. Defined on the basis of their low population prevalence, rare diseases are a heterogeneous group of about 8000 disorders, which are often chronically debilitating, incurable, and, in some cases, life-threatening [33]. At least half of all rare diseases have their onset in childhood [33], meaning that their impact commonly extends beyond the affected individual to involve the entire family unit. Notwithstanding their broad diversity in terms of disease severity and clinical manifestations, families living with rare diseases have been found to experience a range of common problems, directly linked to the rarity of these pathologies [15]. These include, among others, significant diagnostic delays, lack of appropriate information, social isolation, and stigma, as well as difficulties in accessing essential health care services [2, 14, 33].

The way that mothers cope and adjust to their child’s diagnosis is pivotal, not only to the family’s physical and emotional well-being, but also to the child’s own coping and adjustment to the disease [21]. The goal of this qualitative study was to elucidate how the diagnosis of a rare disease, as compared to a non-rare condition, may influence maternal experiences of childhood illness. We recruited mothers living with two substantially different—yet, comparable in various aspects—medical conditions: Bartter syndrome (BS) and celiac disease (CD). BS represents a set of closely related, autosomal recessive renal tubular disorders, characterized by hypokalemia, hypochloremia, metabolic alkalosis, and hyperreninemia with normal or low blood pressure [1]. Its annual incidence in Europe is estimated at 1/1,000,000 [27]. By comparison, CD is a common disorder that can occur in genetically predisposed people where the ingestion of gluten leads to damage in the small intestine [13]. Its prevalence in Europe is close to 1/100 [18]. Yet, both patients with BS and those with CD are reported to face significant delays in getting initially diagnosed. Moreover, given that the two conditions can have similar signs and symptoms, the differential diagnosis of BS frequently includes CD. Both diseases are currently incurable; the treatment, however, of BS is directed at correcting electrolyte disturbances with the use of oral potassium supplements, non-steroidal anti-inflammatory drugs, and, possibly, potassium-sparing diuretics, whereas the treatment of CD typically involves a lifelong gluten-free diet.

## Materials and methods

### Participants

Between February and April 2014, we conducted face-to-face, semi-structured interviews with 26 mothers of children treated in a pediatric hospital in the province of Lecco, Italy. Half of the participants had a child diagnosed with BS and the rest had a child suffering from CD. The mean age of mothers living with BS was 41.6 (SD = 7.3), whereas the mean age of mothers living with CD was 45.8 (SD = 6.5). Tables 1 and 2 present further details on interviewee characteristics.

**Table 1:** Sociodemographics of mothers of children with Bartter syndrome (*n* = 13)

Variable	Category	*n*	%
Age group	25–34	2	15.4
	35–44	7	53.8
	45–54	4	30.8
Nationality	Italian	10	76.9
	Non-Italian	3	23.1
Education	Middle school	2	15.4
	High school/vocational	7	53.8
	University	4	30.8
Working status	Working	8	61.5
	Not working	5	38.5
Marital status	Married	13	100
	Not married	0	0
Number of children	1	2	15.4
	2	9	69.2
	≥ 3	2	15.4
Time since diagnosis^a^	< 5 years	5	38.5
	5–10 years	5	38.5
	> 10 years	3	23.1

^a^Refers to time elapsed since the diagnosis of the first child (in case of mothers having more than one child diagnosed with the disease)

**Table 2:** Sociodemographics of mothers of children with celiac disease (n = 13)

Variable	Category	*n*	%
Age group	25–34	1	7.7
	35–44	5	38.5
	45–54	6	46.2
	55–64	1	7.7
Nationality	Italian	12	92.3
	Non-Italian	1	7.7
Education	Middle school	5	38.5
	High school/vocational	8	61.5
	University	0	0
Working status	Working	6	46.2
	Not working	7	53.8
Marital status	Married	11	84.6
	Not married	2	15.4
Number of children	1	3	23.1
	2	8	61.5
	≥ 3	2	15.4
Time since diagnosis^a^	< 5 years	6	46.2
	5–10 years	3	23.0
	> 10 years	4	30.8

^a^Refers to time elapsed since the diagnosis of the first child (in case of mothers having more than one child diagnosed with the disease)

### Procedure

Participants were recruited with the help of four pediatricians, specialized in treating children with BS and CD. Physicians contacted potential participants by phone and introduced them to the study objectives, while providing them with the contact details of the researchers. They further explained that participation was voluntary and that refusal to participate would in no way affect the child’s care or treatment in the hospital. Mothers willing to participate (all approached but three, who cited lack of time as a reason for non-participation) contacted the researchers and set up a date for the interview. The second author (IV), a social scientist with substantial training in qualitative data collection, conducted most of the interviews, either in participants’ homes or at a private office in the hospital. Prior to each interview, details about how data would be collected, analyzed, and used were discussed, and signed informed consent was obtained. The average duration of the interviews was about 45 min. All interviews, apart from two where both parents were present, were conducted on a one-to-one setting with only the researcher and the participant being present. Data were collected using a semi-structured interview guide (Table 3), developed on the basis of Leventhal’s Common Sense Model of Self-Regulation (CSM) [24]. The CSM has been extensively used in the health literature, as it offers a conceptual framework for examining the perceptual, behavioral, and cognitive processes involved in individuals’ management of current or future health threats [25]. A key construct within the CSM is the idea of illness representations or “lay” beliefs about the illness, which integrate with existing schemata (the normative guidelines that people hold), enabling individuals to make sense of their symptoms, while guiding coping actions [19]. These representations have various attributes, including as follows: (a) identity (the label or name given to the condition and perceptions of associated symptoms), (b) cause (personal ideas about the cause of the condition, which may or may not be biomedically accurate), (c) consequences (individual’s beliefs about the consequences of the condition and how this will impact on them physically and socially), (d) timeline (perceptions about how long the condition might last), and (e) curability/controllability (beliefs about whether the condition can be cured or kept under control and the degree to which the individual plays a part in achieving this) [24].

**Table 3:** Examples of questions included in the Interview guide

Perceived illness identity	
- Can you tell me a little about your child’s condition?	
- How did you find out that she/he is suffering from this disease?	
- How easy has it been for you to understand what is going on with your child and cope with it?	
- How do you usually describe your child’s condition to people around you (e.g., family, friends, school teachers)?	
Perceived illness consequences	
- How would you describe a typical day in your child’s/family’s life? - What are the most difficult things to handle about the disease in everyday life?	
- How has your life changed since your child’s diagnosis?	
Perceived illness causes	
- Do you ever think what might have caused your child’s disease? - Have you discussed your thoughts with your doctor?	
Perceived illness timeline/controllability	
- Do you ever imagine how your child will be in 10/15 years? - Could you share some of your thoughts with me?	

### Analysis

All interviews were audio recorded and transcribed verbatim. Data analysis proceeded concurrently with data collection, using the six-stage process of thematic analysis proposed by Braun and Clark [4]. Although for the development of our interview guide we relied on an empirically tested theoretical model, for the analysis of our data, we sought to employ an inductive approach, identifying themes from participants’ expressed perspectives rather than our own analytic preconceptions. To maximize the validity of our findings, data analysis was carried out independently by two of the authors (EG and IV), who met regularly to discuss the process and reach consensus on emerging themes. Final development of themes was accomplished through joint review and input from the whole team.

### Rigor

Drawing on Lincoln and Guba’s [26] criteria of credibility, transferability, dependability, and confirmability, we employed several techniques for ensuring trustworthiness: analyst triangulation (involving multiple researchers in the analysis process), deviant case analysis (searching for and discussing elements of the data that appeared to contradict emerging patterns or explanations), audit trail (creation of a study database where all steps taken from the start to the end of the project were reported), and “thick” description (a detailed account of our field experiences so that the reader can evaluate the extent to which our findings can be transferred or applied in different settings). We also used the consolidated criteria for reporting qualitative research (COREQ) checklist [32] as a way of facilitating the transparent and comprehensive reporting of all important aspects related to our study design (Supplementary File).

## Results

Three main themes were identified from the analysis of our data and are presented below.

### Disrupted normality and the need to know

The vast majority of informants described the period from the appearance of first symptoms to diagnosis as the most difficult and psychologically painful. The onset of symptoms and the subsequent quest for a diagnosis disrupted participants’ normality, and they used several reference points to express this disruption: (a) the normality they were used to before the disease, (b) other people’s normal family life, and (c) their own ideas and perceptions of how a normal person (or a normal mother) is supposed to act: “Meanwhile, people around us were living a normal life. They had their children, they watched them grow, and we... We were travelling all around Switzerland in search of an answer. And we found all possible people that you can imagine. We even consulted a person who claimed to talk to angels! Can you believe this? But when science is not able to give you the answers you need, you will do everything.” (mother of BS child, 37 years).

Diagnostic delays were common in participants’ narratives, augmenting feelings of insecurity, and uncertainty, whereas frequent medical controls, extensive hospitalizations, and long-distance traveling to access specialized centers made normal family life impossible: “I was at the hospital day and night, while my husband was at home taking care of our older son. He was 3 years old back then and he had to understand that his younger brother was at the hospital and his mother had to be there with him…” (mother of BS child, 48 years). The longer and more complicated the diagnosis process became, the more stressful this period was perceived to be and the stronger participants felt the need to know.

### Reconstructing normality

Reaching a diagnosis, although causing an initial shock reaction, was mostly experienced as a relief (especially by mothers who had endured long diagnostic delays). The diagnosis was perceived as the first step towards treatment, a way of reducing child’s suffering, as well as a turning point that could enable families to start adjusting to their new normality: “I had reached a point where... I remember, I didn’t even want to bathe her. She had this swollen abdomen, these extremely skinny legs. So, yes, I was happy because we finally knew what the problem was and we could do something about it.” (mother of CD child, 48 years). The process of normality reconstruction involved a redefinition of normality as incorporating the illness and practically implied, besides reorganizing family life based on the needs of the sick child, gaining a sense of control over the disease.

Yet, this process appeared to be very different between the two groups of participants and strategies that facilitated disease management and acceptance among mothers living with CD, namely access to appropriate information, social support, and personal contact with comparison others, emerged as important stressors for mothers living with BS. Specifically, mothers caring for a CD child talked extensively about the support provided by the Italian Celiac Association: “The information they gave us was really useful, especially at the beginning that we had to reorganize our habits. They sent us a handbook that contained information on gluten-free foods… Now they have also created a smartphone app!” (mother of CD child, 43 years). Furthermore, having the possibility of personal contact with other patients and their families appeared to be extremely beneficial, as it provided emotional and practical support (e.g., exchange of gluten-free recipes, suggestions about restaurants), while reducing feelings of social isolation.

Although mothers living with BS had sought to employ the same strategies to cope with the disease and adjust to their new normality, the rarity of the condition had impeded the success of their actions. Participants reported that reliable information was scarce and/or difficult to find, whereas social support was minimal. Bureaucratic problems were frequently stated, as well as difficulties in navigating through the social and health care system. The most important stressor, however, was the lack of personal contact with comparison others: “For other rare diseases, there are groups, but we don’t know anyone else. And we miss this. I mean, having the opportunity to compare ourselves with other families... Maybe it would also give us a bit more courage to think: look, they made it!” (mother of BS child, 47 years). Personal contact with comparison others was seen as fundamental for various reasons: having someone that “speaks the same language,” exchanging information about everyday problems, feeling less alone, and reducing uncertainty caused by the disease and forming an idea of what to expect from the future.

Faced with a fragmentation of their initial efforts, normality reconstruction appeared to be for mothers living with BS a much more complex, demanding, and time-consuming process, which usually came years after the diagnosis, when the child’s condition was perceived to be more stable and they felt more confident in handling the disease. Yet, not knowing how the disease will progress created a fragile sense of control, which could be easily shattered in case of an unexpected event: “Now we are coping well with it. But there are times... For instance, the doctor called us last week and told us that he wants to see us... And I immediately started: “Why? What happened? Did you see something in the exams?” I mean, you always have this... There is always this shadow that follows you.” (mother of BS child, 43 years).

### Acting “normal”

Mothers’ ability to convey their new normality both to external others and to the child formed an integral component of normality maintenance. All participants felt the need to ensure that people around them are somehow aware of the problem and know what to do in case of an emergency. A key difference, however, between the two groups was that mothers living with CD openly named the disease to their social environment, as “nowadays everyone is aware of celiac disease,” whereas mothers living with BS usually avoided using “medical terms” and, instead, tended to provide an oversimplified description of main disease symptoms. The avoidance of the terms “rare disease” and “syndrome” was mostly linked to participants’ willingness to protect the child from stigma and panic-like reactions: “When you say: “he has a rare disease,” people look at you like he has something terrible! Maybe it’s the word “rare,” it alerts people.” (mother of BS child, 47 years). Yet, the complexity of the disease posed an additional barrier to diagnosis disclosure. In particular, the distinction between lay and medical knowledge was evident in participants’ accounts, with some mothers reporting not feeling knowledgeable enough to adequately explain the disease to others, while others feeling skeptical about the degree of medical information that people around them would be able to understand.

Not making the child feel “sick,” that is, limited in any way by the disease or different from others, was perceived as of utmost importance, and mothers reported using specific terminology when talking about the disease (e.g., the term “problem” was typically preferred over the terms “disease” or “illness”): “He has to live peacefully, he is just a kid! He doesn’t need to think all the time that he is sick. He is like the others and he has to live like the others. He has this problem and he needs to take potassium… I explained to him that this is a thing that our body produces… He has to take it because his body doesn’t produce it. But it’s not a drug or anything.” (mother of BS child, 38 years). In the same way, informants narrated how they tried to avoid situations where the child’s normalcy would be threatened: “Yesterday they went on a school trip and everybody went to this amazing ice-cream shop… But he knows that, in cases like this, he should go and buy an ice-lolly instead. We always propose alternatives, so that he doesn’t feel like there are things that he cannot do…” (mother of CD child, 41 years).

Yet, acting “normal” was not always easy and several factors appeared to challenge participants’ efforts to behave in a “normal” way. In both groups, a dichotomic conceptualization of context as *inside* vs. *outside the home* was evident, with mothers feeling in control of the disease inside the home, while fearing that the worst consequences of the disease might be experienced outside the home: “The problem is when we go out. Tonight, for example, he has a dinner with the team… It’s really the outside, away from home, this is the difficult part.” (mother of CD child, 49 years). Moreover, in the case of mothers living with BS, the severity of disease symptoms and the degree of involvement in complex every-day treatments and medical decision-making constituted an additional factor challenging the “normal” family life: “Sometimes, we don't feel like parents, we feel like home doctors. With our daughter we also got to insert the nasogastric tube, it’s something we did once, but we got to do that, too...” (mother of BS child, 32 years). Treatment administration was often seen as a time-consuming and unpleasant procedure, which affected the relationship with the child and did not leave space for a “normal” family life: “The lunch can last for two hours... The lunch and then the medicines, and then we have one hour of free time, and then it is time for the afternoon snack. Another hour for this, then the medicines again.” (mother of BS child, 41 years).

## Discussion

Qualitative research can provide a powerful tool for understanding biomedical reality in terms of illness experience and its sociocultural context [6, 17, 30]. Yet, qualitative investigations are still scarce in the field of rare diseases, although challenges to recruiting adequate sample sizes for ensuring the validity of quantitative findings have been frequently reported [2, 14, 23, 28]. This qualitative study provides an in-depth understanding of the impact of a rare disease diagnosis on mothers’ experiences of illness and illness management behaviors. Our results, based on qualitative interviews with mothers living both with a rare disease and with a non-rare condition, offer valuable insights into the complexity of conceiving normality in the context of a childhood chronic illness, while highlighting important differences between the two groups in terms of processes.

Consistent with the conceptualization of chronic illness as a “particular type of disruptive event” [5], participants in our study described how the appearance of first symptoms and the subsequent quest for a diagnosis disrupted the structures of everyday life and its taken-for-granted features, marking the end of their previous known normality. The diagnosis per se was seen as a turning point that could enable families to start adjusting to their new normality. The process of normalization, as a form of coping with chronic illness, has been studied in various contexts, including as follows: rheumatoid arthritis patients [31], kidney transplant patients [3], cancer patients [12], and families of children with osteogenesis imperfecta [9]. Knafl and Deatrick [22], focusing on families’ attempts to respond to disability, found that there is an implicit moral acceptance of normalization as good. The normalization of illness has been shown to be feasible through a previous experience of the illness [16], association of older age with illness [29], and self-association with one’s community (e.g., other patients) [7]. Similarly, our results suggest that personal contact with comparison others, access to appropriate information, and the presence of a supportive social network had facilitated the process of normality reconstruction among mothers living with CD, while hindering the same process for mothers living with BS.

Both engaging in behavior that demonstrates normalcy to others and developing a treatment regimen that is consistent with normalcy have been identified as key elements in the normalization process of families living with chronic conditions [10]. In our study, acting “normal” emerged as a key priority for all participants. Yet, even in this case, barriers linked to the rarity of the condition were evident. Specifically, although mothers of children with CD openly named the disease to their social environment, mothers of children with BS chose not to reveal the diagnosis. Instead, explanations provided typically focused on emphasizing the child’s normalcy, but with some exceptions (e.g., “He is like all other children, just a bit shorter”). In this context, words like “rare” and “syndrome” were seen by most informants as potential barriers to demonstrating normalcy. Furthermore, in line with studies underlining the centrality of the treatment regimen to normalization [8, 9, 20], we found that involvement in complex every-day treatments frequently challenged the efforts of mothers living with BS to view both the child and their life as normal.

To the best of our knowledge, this is the first study offering an insight into the experiences of mothers caring for a child with BS, while addressing the challenges of normalization for families living with a rare disease. Among the main strengths of this work are that we employed a rigorous qualitative approach, we relied on a sample which allowed for data saturation, and we used “thick description,” namely a detailed account of our field experiences, to maximize the transferability of our findings to other contexts or settings. Yet, the fact that all of our participants were recruited from the same hospital could be a potential limitation. Likewise, we cannot exclude the possibility of recall bias, as mothers often commented on longer term events rather than their current experiences of living with the disease. Longitudinal qualitative research is needed to overcome the limitation of retrospective data by exploring families’ experiences at different time points of the healthcare trajectory.

## Conclusions

Clinicians and policy-makers require an understanding of the factors that facilitate and hinder normalization in families living with rare diseases. Our results suggest that maximization of emotional and instrumental social support, through provision of appropriate information or establishment of disease-specific support groups, could greatly contribute to rare disease families’ efforts to cope with childhood illness and regain a sense of normality.

## Electronic supplementary material

ESM 1 (DOCX 17 kb)

## Abbreviations

BS: Bartter syndrome
CD: Celiac disease
